# RA.DI.CA. Splint Therapy in the Management of Temporomandibular Joint Displacement without Reduction

**DOI:** 10.3390/jpm13071095

**Published:** 2023-07-03

**Authors:** Carlo Di Paolo, Erda Qorri, Giovanni Falisi, Roberto Gatto, Sergio Rexhep Tari, Antonio Scarano, Sofia Rastelli, Francesco Inchingolo, Paola Di Giacomo

**Affiliations:** 1 Department of Oral and Maxillo-Facial Sciences, Sapienza University of Rome, 00185 Roma, Italy; carlo.dipaolo@uniroma1.it (C.D.P.); p.digiacomo@uniroma1.it (P.D.G.); 2 Department of Dentistry, Faculty of Medical Sciences, Albanian University, 1001 Tirana, Albania; e.qorri@albanianuniversity.edu.al; 3 Department of Life Health and Environmental Sciences, University of L’Aquila, 67100 L’Aquila, Italy; giovanni.falisi@univaq.it (G.F.); roberto.gatto@univaq.it (R.G.); sofia.rastelli@graduate.univaq.it (S.R.); 4 Department of Innovative Technology in Medicine and Dentistry, University of Chieti-Pescara, 66100 Chieti, Italy; sergiotari@yahoo.it; 5 Department of Interdisciplinary Medicine, University of Bari “Aldo Moro”, 70121 Bari, Italy; francesco.inchingolo@uniba.it

**Keywords:** occlusal splints, arthralgia, temporomandibular joint disorders, magnetic resonance imaging

## Abstract

Background: The purpose of this study is to report clinical and instrumental changes after RA.DI.CA splint therapy for temporomandibular joint disc displacement without reduction. Methods: Subjects affected by disc dislocation without reduction were recruited between July 2020 and May 2022 based on inclusion and exclusion criteria and treated with RA.DI.CA. splints over a period of 6 months. Clinical data were collected at each phase of the study (T0, T1, T2). Magnetic resonance imaging and electrognathography data were recorded at the beginning (T0) and at the end (T2) of the study. ANOVA with post-hoc contrasts was performed to assess differences in outcome measures over time. The Wilcoxon test was used to evaluate changes in disc-condyle angle between before- and after-treatment MRI. A two-tailed value of *p* < 0.05 was regarded as significant. Methods: Ten patients completed the study. There were statistically significant differences over time for arthralgia, headache, neck pain, and mouth opening. Disc recapture and an improved quality of mandibular movement were recorded in 70% of subjects. The clinical and instrumental improvements are probably due to the orthopedic action of RA.DI.CA splint treatment, which allows for a greater degree of joint mobilization. Conclusions: The purpose of this therapy is to recover the disc position if possible and achieve an adequate joint functional adaptation that avoids the progression of the structural damage and the recurrence of symptoms.

## 1. Introduction

Pathologies affecting the temporomandibular joint may be related to structural defects and/or functional relationships between its components [1,2]. Clinical manifestations ranging from disc dislocation with reduction to disc dislocation without reduction and osteoarthrosis as reported in DC/TMD [3,4]. Disc dislocation without reduction (DDwoR) is characterized by permanent antero-medial disc displacement resulting in the functional limitation of the mandible, also called “closed lock”, and represents the end stage of a condyle-disc dysfunction after a history of joint noises [5,6].

Closed lock is acute when the onset is abrupt and persists for no more than 20–30 days; sporadic when the patient reports only a few episodes over a very long period of time, i.e., no more than three episodes in 1 year; recurrent when the patient reports episodes occurring with a certain frequency; intermittent when the patient reports daily episodes; and chronic when hypomobility has been continuous for more than 30 days. It can be distinguished as “permanent” when it is subsequent to an acute episode without symptom remission or “terminal” when it derives from recurrent or intermittent manifestations [7]. Disc displacement can be diagnosed by clinical investigation and imaging methods. The recent improvement of diagnostic imaging has significantly increased the possibility of a better understanding of temporomandibular dysfunctions (TMDs) [8]. Magnetic resonance imaging (MRI) of the TMJ can provide indispensable information about the position [9], signal intensity [10], morphology and structure of the disc [11], quantity of the synovial fluid [12], condition of the bone [13], posterior attachment and retrodiscal tissues [14], bone marrow [15], and scar tissues [16]. Furthermore, MRI analyzes the relationships between joint structures, and, above all, it is the only method that can give information about the position of the joint disc. These MRI characteristics are essential for diagnosis and treatment planning.

Therefore, MRI should be used for pre- and post-treatment assessments of the biomechanical aspects of joint structures. From a functional point of view, a more objective recording of clinical data can be achieved by electrognathography, which evaluates both antero-posterior and lateral mandibular movement, measuring the mandibular opening, the lateral deviation, the spontaneous movement, and the relaxing habitual position of the mandible. It is mainly used for the objective analysis of the mandibular movements [17]. Several kinds of treatment protocols have been proposed for its resolution, such as physiotherapy, drugs, occlusal splints, and surgical options [5,18,19]. Regardless of the method used to solve the joint lock, the immediate treatment aims at recapturing the articular disc. If that is not possible, treatments should be targeted at improving mandibular function and remodeling the soft tissues of the temporomandibular joint [20]. Among the conservative splint therapies, the patented RA.DI.CA. splint (acronym for Rampello–Di Paolo-Cascone License No. 91-000571) is able to diminish acute painful symptoms in favor of a specific orthopedic rehabilitation. As stated in previous studies [21,22], the major effectiveness of the RA.DI.CA splint compared to other ones is due to greater distraction and orthopedic action, with the aim of mobilizing temporomandibular joints, through a downward and forward movement of the mandibular condyles, which also allows for the restoration of muscular functionality. Recent studies describe a detailed DDwoR protocol combined with pharmacological and physical treatments customized considering the individual characteristics of the subject [21]. Previous studies investigated its clinical effectiveness and compared it with other occlusal devices [22]. In the scientific literature, as for the goals of mandibular joint locking therapy, the need for disc recapturing is still debated in terms of functionality and symptom improvement. Furthermore, most of the splints used for DDwoR treatment have a passive action of stabilization without joint mobilizing or disc recapturing. The hypothesis of this study is to verify if joint disc recapturing is possible through a RA.DI.CA splint and identify the possible clinical implications of this treatment.

The purpose of this research is to report clinical changes after DDwoR treatment with a RA.DI.CA splint, also evaluated with MRI and electrognathography.

## 2. Materials and Methods

The authors designed an open label and single-arm pilot trial. The present investigation was started in July 2020 and finished in May 2022, and it was conducted in the Gnathology Unit Service of the Integrated Head and Neck Care of the Department of Policlinico Umberto I, “La Sapienza” University in Rome. The clinical research received the approval of the Institutional Ethics Committee of La Sapienza University (Prot.no. 349 24/10/2018) and the Institutional Ethics Committee of Albanian University (Prot No. 139—22/02/2022). The clinical study was registered in the ISRCTN registry (Prot. N. 77777174). All subjects involved in the study signed informed consent.

The clinical trial was performed in accordance with the Principles of the Declaration of Helsinki for research clinical trials on humans. Informed consent was obtained from all subjects involved in the study.

### 2.1. Participants and Sampling

The expected sample size, according to Birkett and Day [23], for an internal pilot study is 10 subjects. The subjects included in the present investigation were visited by experts and calibrated examiners in accordance with the DC/TMD Criteria during a study period of 15 months.

The following inclusion and exclusion criteria were considered in the present study:

Inclusion criteria: (a) subjects >18 years old; (b) diagnosis of jaw functional limitation associated with unilateral disc displacement with no reduction for <6 months, verified with MRI in accordance with the RDC/TMD and DC/TMD criteria.

Exclusion criteria: (a) jaw function limitation associated with other diseases including connective tissue pathologies and scleroderma, fracture and trauma, deformities, cancers, TMJ ankylosis, and muscular locking; (b) patients under conservative therapies including physical rehabilitation.

The DDwoR diagnosis was given considering the typical pathological signs and symptoms, including the mandible deviation towards the affected side associated with the mouth opening movement, negative evidence of end-feel testing, limitations of the mouth opening, and pain associated with the affected joint compared to less frequent pain on the contralateral side.

Diagnosis was also completed by MRI. MRI was performed with a 1.5 tesla machine. The investigations were all performed with the spin echo acquisition technique according to oblique and coronal sagittal scanning planes with dependent T1 and T2 sequences in conditions of habitual occlusion and maximum mouth opening. The following MRI parameters were analyzed in order to evaluate qualitative joint features before and after treatment: (a) articular effusion; (b) disc deformity; (c) lateral or medial displacement; and (d) retrodiscal tissue alterations. As for a quantitative analysis, the authors measured the disc-condyle angle theta (θ) as described by Drace and Enzmann [24], selecting the sagittal slice perpendicular and through the center of the horizontal long axis of the condyle. The normal values for angle θ range from −15° to 15°, and an angle θ of >15° indicates the presence of anterior disc displacement [25].

### 2.2. Treatments and Protocols

**Figure 1:**
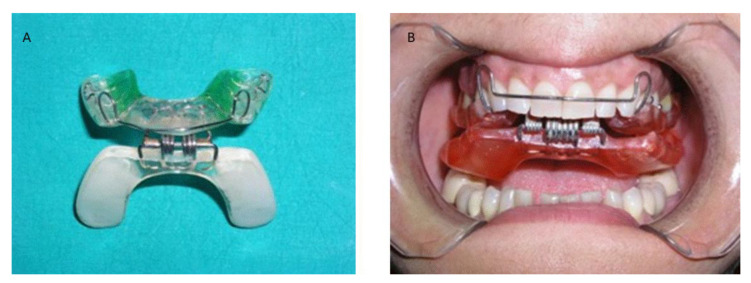
(**A**,**B**). RA.DI.CA. splint and its parts.

**Figure 2:**
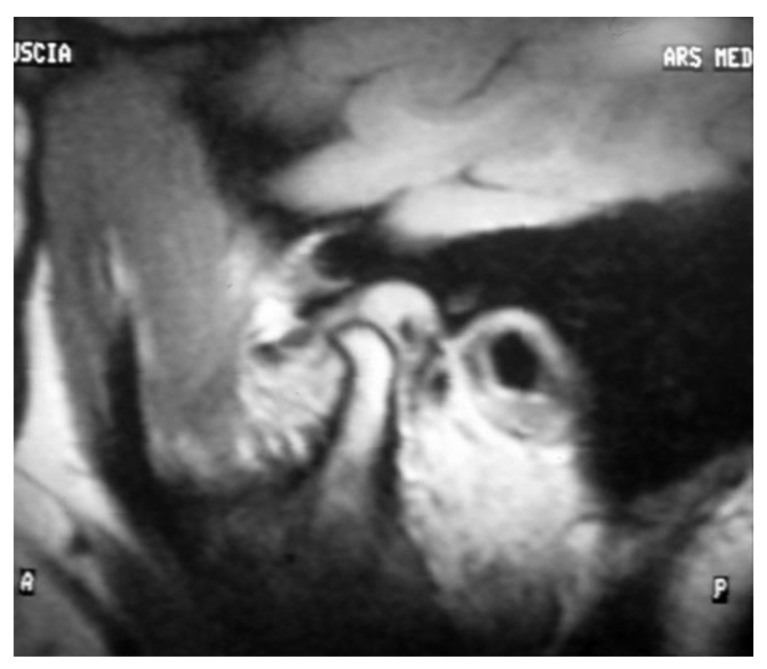
Magnetic Resonance Image before treatment T0. The MRI shows a joint effusion, degenerative bone alterations, disc displacement and remodeling.

The RA.DI.CA. splint is a device characterized by the following (Figure 1 and Figure 2):

- An upper plate made with heat-cured acrylic resin (1),
- A lower plate made with heat-cured acrylic resin (2),
- An anterior hinge (3),
- A total of vestibular springs obtained by an orthodontic wire (4),
- A minimum of 2 Adams clasps with/without 2 ball clasps (5), and
- A steel arch at the level of the vestibular side (6).

The upper plate is provided by a total of 2 surfaces. The inner part is known as the “occlusal/palatal” component, and it is able to fit the upper teeth masticatory side and the 2/3 anterior palatal vault part. The outer part is smooth and faces the lower plate. The two Adams clasps are positioned on the 1.6 and 2.6 teeth, while the balls are positioned at the level of the interdental region between the 1.4–1.5 and 2.4–2.5. The upper plate is also characterized by stabilizing capability, due to the mucosal and dental anchorages.

The lower plate is made using a horseshoe profile with a total of two different smooth surfaces. The first part is made in contact with the upper plate outer component, while the second part is in contact with the lower teeth masticatory surfaces. In addition, the upper plate should be maintained with consideration of the Spee and Wilson physiological curves.

The two plates are connected by a front hinge positioned at the level of the incisor part, and the two vestibular springs positioned on the left and right sides connect at the level of the canine-premolar region (Figure 1). This device structure is able to obtain an improved lower plate elastic strength during functional movements and mouth closure. The springs used can have hard or soft strength depending on the patient’s individual characteristics. The functional action is obtained through a push mechanism at the level of the posterior region of the mandible arch. It can induce a downward and forward movement of the mandible condyle process. In addition, the device aims to enable recovery of the correct functional and anatomical relationship between the articular disc and mandible condyle process, while in the case of a more severe clinical condition and a non-predictable complete recovery, the device aims to provide jaw function rehabilitation and a significant reduction of acute pain. The clinical protocol was conducted by instructing the patients to wear the device for 2 h during the daytime and during the night, with periodic follow-up appointments. Daytime therapy use is dedicated to active exercises and gymnastic where patient does opening and contra-lateral movements in order to improve the functional recovery of the affected side [21]. No additional drug or manual treatment was applied for either of the groups.

### 2.3. Study Phases

The clinical study was conducted with a total of three different phases:

T0 = recruitment period
T1 = 4 weeks from the start of therapy
T2 = 6-month follow-up

### 2.4. Study Variables

**Figure 3:**
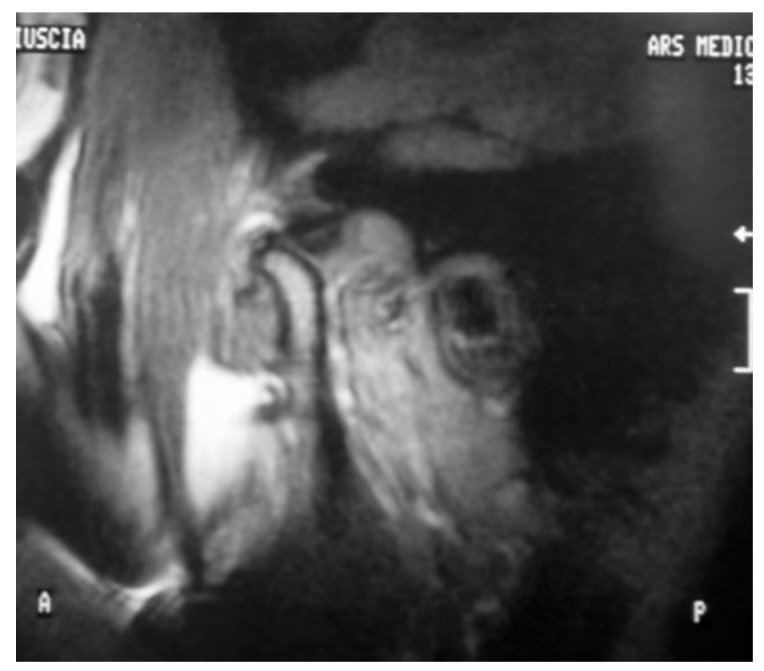
Magnetic Resonance Image after treatment T2. An improvement in disc position is observed.

**Figure 4:**
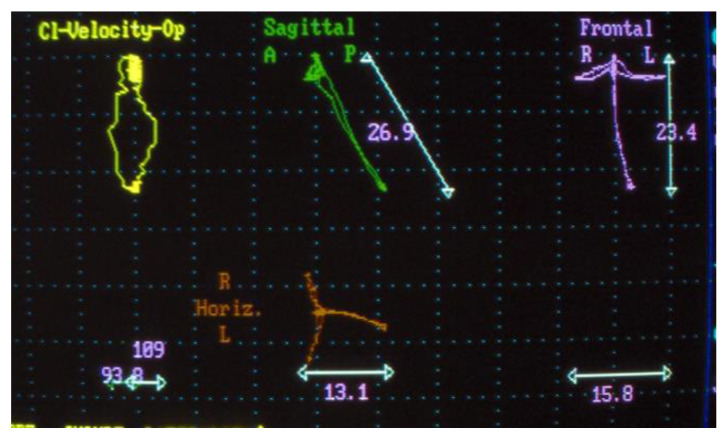
Electrognathography before T0.

**Figure 5:**
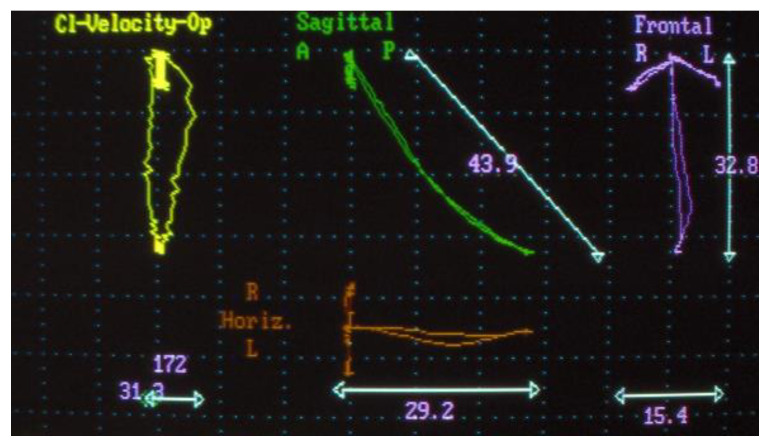
Electrognathography after T2.

1. Demographic independent variables: gender, age, marital status, and job.
2. Occlusal and dental variables: occlusal and skeletal class, teeth formula, occlusal alterations, incisal guide, teeth loss, and parafunctions. These parameters were collected based on the clinical examination and standard lateral radiograph and dental orthopantomography.
3. Medical history, including trauma, TMJ clicking, and correlated symptoms.
4. Pain characterization: TMJ-related pain including arthralgia and muscle pain, headache, familiar related pain, neck pain, and emotional stress, following a verbal numeric scale (VNS) classification conducted with the patients at all follow-up timepoints.
5. Functional variables: maximum mouth opening and lateral excursions in mm, with measurements taken with the patients at all follow-up timepoints.
6. Days necessary for the closed lock and degree of symptom resolution.
7. Residual clicking after the treatment and occlusal change perception of the patients.
8. MRI evidence, including degenerative bone alterations, disc displacement and remodeling, and joint effusion at T0 and T2 (Figure 2 and Figure 3).

- Electrognathographic parameters. Movement trajectory for opening, closing, and masticatory cycle with elastic hard bolus (Figure 4 and Figure 5). All patients underwent mandibular functional evaluation using the electrognathograph (BioEMG, BioResearch, Inc., Milwaukee, WI, USA) according to our protocol, which included:
- Slow mandibular opening and closing movements starting from the position of maximum intercuspidation.
- Right and left lateral movements starting from the maximum occlusion.
- Chewing for about 1 min of the elastic hard bolus.

### 2.5. Clinical Calibration

In order to establish a degree of agreement in the judgement made based on the qualitative assessment of a patient by several practitioners, so that the diagnosis and the treatment are not operator dependent, an estimate is usually made by means of the Cohen’s kappa coefficient. In the present study, the authors sought to overcome intra and inter operator variability by basing their assessment more on objective quantitative than qualitative parameters.

### 2.6. Statistical Analysis

Repeated measures of ANOVA with post-hoc contrasts were performed to assess differences of outcome measures over time. The Wilcoxon test was applied to investigate the changes in disc-condyle angle between before- and after-treatment MRI. A two-tailed value of *p* < 0.05 was considered as significant. All analyses were performed with JASP Version 0.8.0.1, downloadable at https://jasp-stats.org/download/, accessed on 1 June 2023. Results were controlled using SPSS 24, and no discrepancy was found.

## 3. Results

**Figure 6:**
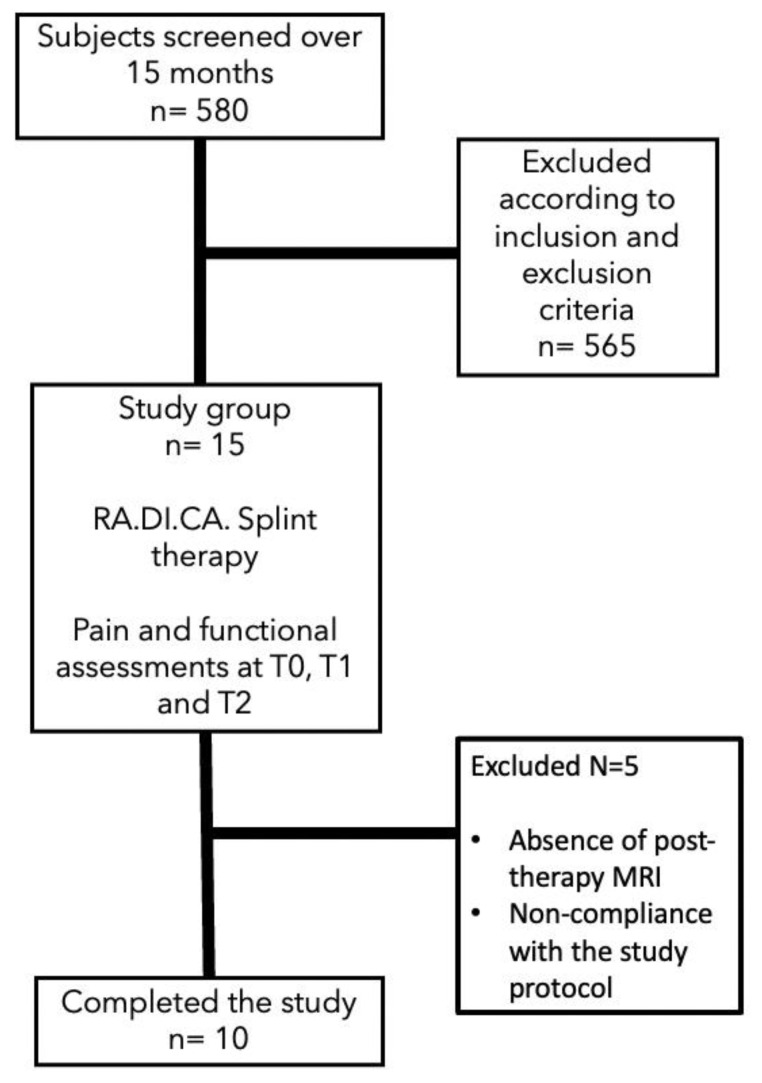
Consort flow chart.

The study flow chart has been described according to the CONSORT guidelines and reported in Figure 6.

### 3.1. Descriptive Statistics

The population sample included a total of 10 subjects (two male and eight female), with mean age 41.10 ± 11.00. Six subjects (60%) had class I dental occlusions, and four subjects (40%) had class II dental occlusions. Four subjects (40%) had a normal incisal guide, four subjects (40%) had a deep one, and two subjects (20%) had a vertical one.

All the patients referred to previous joint noises, two subjects (20%) referenced previous traumas, and eight subjects (80%) noted parafunctions.

At the baseline, the evidence of the VNS scale reported strong pain characterized by a values range between 50 and 80 of all clinical parameters considered, including arthralgia, headache, and neck pain. On the other hand, mouth opening measurements had an average value below the normal range of 45 ± 5 mm. As for MRI parameters, before-treatment qualitative and quantitative analyses are reported below in Table 1.

As for electrognathography, the opening movement in patients affected by disc displacement without reduction can be traced back to the framework of a limitation of the maximum opening with mandibular deviation towards the affected side.

Lateral movement contralateral to the affected side is reduced.

The distribution of chewing cycles remains on the affected side.

Significant differences over time were recorded in all the clinical parameters observed, as shown in Table 2.

### 3.2. Inferential Statistics

**Figure 7:**
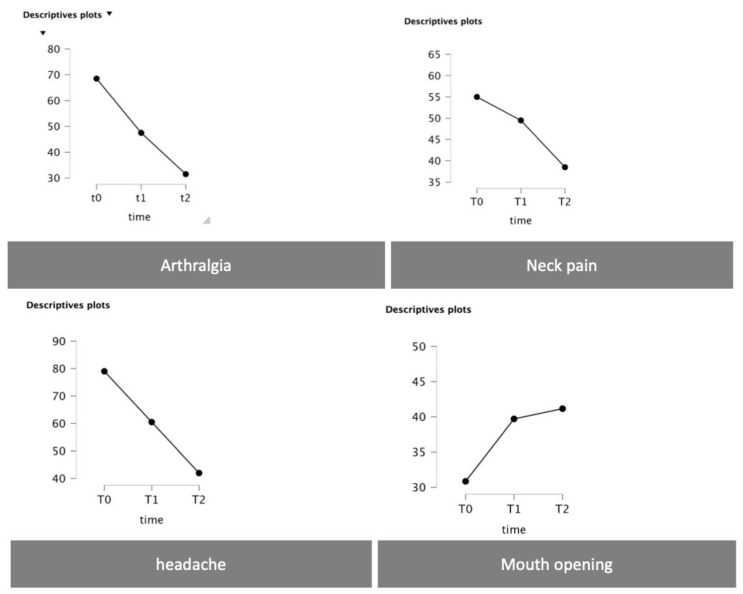
Plots with the time trend as for arthralgia, neck pain, headache, and mouth opening.

Statistically significant differences over time for both arthralgia (*p* < 0.001) and headache (*p* < 0.001) were reported. Neck pain was reported to have a significant difference considering the follow-up periods with comparisons T0–T2 (*p* < 0.001) and T1–T2 (*p* < 0.001). The same evidence was also reported considering the mouth opening at the timepoints comparisons T0–T1 (*p* < 0.001) and T0–T2 (*p* < 0.001) (Figure 7 and Table 3).

As for MRI, disc recapture was recorded in seven patients (70%); three subjects (30%) did not report changes in the relationship between joint structures, as reported in Table 4.

The Wilcoxon test reported a statistically significant difference of the disc-condyle angles measurement registered before and after treatment (*p* = 0.036).

During the electrognathographical evaluation, five subjects reported a deviation to the right side and five to the left one. After therapy, seven subjects reported a change in the pattern of mandibular movement from a deviation to a deflection; three subjects reported no change in the quality of movement and were the same ones who did not report disc recapture.

## 4. Discussion

The clinical results obtained with RA.DI.CA. have already been reported in other papers [21,22], where it proved to perform better in comparison with other splints mainly used in the past. Instead, the aim of this study is to also report the functional and anatomical relationships between the TMJ structures before and after treatment, as recorded by MRI and electrognathography.

In fact, in the previous studies in the literature, the primary goal of DDwR treatments has always been the recovery of symptoms and function, regardless of what changed within joint structures. This was achieved by devices that would allow muscle relaxation and soft tissue distraction [19]. The rationale was based on the fact that functional recovery and the reduction of acute pain symptomatology, at least in the short-term, were independent from changes in disc position [26]. This theory is endorsed by a previous study of Ohnuki et al., which compared four treatments of DDwoR using clinical and MRI records before and after therapy. Although the recovery in function and symptomatology included almost the entire sample, joint disc recapture occurred in only 10% subjects undergoing treatments such as splint therapy, pumping manipulation, arthrocentesis, and arthroscopic surgery, as confirmed by MRI [19]. Except for this study, there are no studies in the scientific literature that evaluate the position and morphology of joint disc during MRI after DDwoR treatment.

This study aims to show how, in addition to facilitating functional and symptomatic recovery, this device can seek congruence in the condylar-discal relationship by recapturing the joint disc, as shown through MRI. Disc recapture occurred in seven out of 10 subjects. The importance of trying to achieve joint disc recapture lies in the concept that when possible, it prevents subsequent and further structural changes and stabilizes function and symptomatology over time. In fact, for example, the “spontaneous” improvement in the mandibular opening indicates that the disc has become more displaced and had a greater deformity. Disc recapture is not possible in all the patients due to “advanced” changes in morphology and position of the disc, but, even if recapturing was not possible, adequate joint functional adaptation was always achieved with an almost complete remission of symptoms. The results obtained with the device represent the cut-off that allows us to determine whether the patient should undergo surgical treatment to solve the dysfunction when both symptoms and functionality do not recover. Furthermore, even if the patient does not wish to undergo surgery, the device may have superior performance compared to other devices.

The study also showed an improvement of headaches and neck pain, which are defined as comorbidities of temporomandibular joint disorders. Subjects with TMJ disorders often complain of headaches and neck pain. The main hypotheses relate to the neuroanatomical connection between the structures of the cranio-cervico-mandibular district and the convergence of nociceptive inputs at the trigeminal level [27,28,29]. In this case, as reported in other studies [30,31], headaches and neck pain are probably more related to the negative adaptation of cranio-cervico-mandibular muscle chains than to the joint dysfunction. Through the resolution of the mechanical lock and the improvement of masticatory muscle function, headaches and neck impairment seem to improve as well, as also reported in other trials where the goal of therapy was muscle relaxation [32,33]. The authors did not report masticatory muscle pain because the arthrogenic pain was prevalent, but the majority of patients reported moderate masticatory muscle pain.

Ultimately, the type of pain treated through splint therapy and, in this case, with RA.DI.CA is a nociceptive pain related to biomechanical dysfunction. In fact, chronic joint pain due to an inveterate joint disease follows other pain transmission criteria also related to central sensitization mechanisms, with a negative impact on therapy outcomes.

However, it would be appropriate to carry out an MRI pre and post comparison with another device to demonstrate how this effect cannot be achieved with a standard device. As for electrognathographical records, seven out 10 subjects showed an improved quality of mandibular movement with a transition from a deviation, sign of a disc displacement without reduction, to a deflection, sign of a disc displacement with reduction.

The limitations of the study are the small sample size and the lack of a control group with other occlusal splints. In fact, it is necessary to verify the presence or absence of changes in the spatial relationship of the condyle-disc unit and in the pattern of mandibular movements in subjects who underwent other types of treatment. Therefore, the future direction is to overcome these limitations and, above all, to establish more frequent long-term follow-ups that assess the actual need to attempt the recovery of disc position or at least ensure more effective joint mobilization in the treatment of DDwoR.

## 5. Conclusions

The encouraging results obtained in the previous studies have led to further investigation of the clinical implication of the use of a RA.DI.CA splint so that the peculiarities of this device could be assessed through a more objective perspective such as an instrumental evaluation. As for the sample analyzed, the current study showed that almost all of the subjects who underwent treatment with a RA.DI.CA splint had an improvement in mandibular functionality and a recovery of disc position, in support of positive changes also in terms of symptomatology. Further investigations are required with a larger sample and long-term follow-ups.

## Figures and Tables

**Table 1:** Before-treatment qualitative and quantitative MRI analyses.

	Qualitative Evaluation				Quantitative
	Lateral or Medial Displacement/Anterior	Disc Deformity	Articular Effusion	Retrodiscal Tissues	Disc-Condyle Angle Theta
Subject 1	Antero-medial	no	Yes	none	23°
Subject 2	Antero-medial	no	Yes	none	24°
Subject 3	Anterior	no	Yes	none	22°
Subject 4	Anterior	no	no	none	30°
Subject 5	Anterior	yes	no	none	25°
Subject 6	Antero-medial	yes	Yes	none	25°
Subject 7	Anterior-medial	no	no	none	20°
Subject 8	Antero	yes	no	none	35°
Subject 9	Antero-medial	no	yes	none	19°
Subject 10	Antero-medial	yes	no	none	30°

The disc-condyle angle value was outside the normal range (22.55 ± 5.22).

**Table 2:** Clinical characteristics at T0, T1, and T2.

Clinical Parameter	T0 (Mean ± SD)	T1 (Mean ± SD)	T2 (Mean ± SD)
Arthralgia	69.00 ± 17.28	39.33 ± 4.84	14.80 ± 3.72
Headache	83.00 ± 13.37	46.00 ± 3.69	14.66 ± 3.46
Neck pain	57.00 ± 24.51	37.33 ± 5.7	24.00 ± 5.08
Maximum mouth opening	30.40 ± 3.47	43.13 ± 0.84	44.66 ± 0.791

**Table 3:** Repeated measures of ANOVA with time as the within-subject factor. Level of significance *p* < 0.05.

Parameter	F	*p*-Value	Post-Hoc Test *p* Holm Value		
			T0→T1	T0→T2	T1→T2
Arthralgia	60,021	<0.001	<0.001	<0.001	<0.001
Headache	25,728	<0.001	0.002	<0.001	0.002
Neck pain	14,459	<0.001	0.086	<0.001	0.002
Mouth opening	55,471	<0.001	<0.001	<0.001	0.179

**Table 4:** After-treatment qualitative and quantitative MRI analyses.

	Qualitative Evaluation				Quantitative
	Lateral or Medial Displacement/Anterior	Disc Deformity	Articular Effusion	Retrodiscal Tissues	Disc-Condyle Angle Theta
Subject 1	Normal	no	no	None	−10°
Subject 2	Normal	no	no	None	8°
Subject 3	Normal	no	no	None	5°
Subject 4	Anterior-medial	no	no	None	30°
Subject 5	Normal	yes	no	None	−10°
Subject 6	Normal	yes	no	None	−6°
Subject 7	Anterior-Medial	no	no	None	20°
Subject 8	Normal	yes	no	None	−11°
Subject 9	Normal	no	no	None	5°
Subject 10	Anterior	yes	no	None	30°

## Data Availability

All experimental data to support the findings of this study are available by contacting the corresponding author upon request.

## References

[1] Dowrkin S.F. (1992). Research Diagnostic Criteria for Temporomandibular Disorders: Review, Criteria, Examinations and Specifications, Critique. J. Craniomandib. Disord..

[2] D’Attilio M., Scarano A., Quaranta A., Festa F., Caputi S., Piattelli A. (2007). Modification of Condyle Anatomy Following a Monolateral Bite Rise: A Histological Study in Rat. Int. J. Immunopathol. Pharm..

[3] Schiffman E., Ohrbach R., Truelove E., Look J., Anderson G., Goulet J.-P., List T., Svensson P., Gonzalez Y., Lobbezoo F. (2014). Diagnostic Criteria for Temporomandibular Disorders (DC/TMD) for Clinical and Research Applications: Recommendations of the International RDC/TMD Consortium Network* and Orofacial Pain Special Interest Group. J. Oral. Facial Pain Headache.

[4] Falisi G., Gatto R., Di Paolo C., De Biase A., Franceschini C., Monaco A., Rastelli S., Botticelli G. (2021). A Female Psoriatic Arthritis Patient Involving the TMJ. Case Rep. Dent..

[5] Miernik M., Więckiewicz W. (2015). The Basic Conservative Treatment of TMJ Anterior Disc Displacement Without Reduction—Review. Adv. Clin. Exp. Med..

[6] Macrì M., Murmura G., Scarano A., Festa F. (2022). Prevalence of Temporomandibular Disorders and Its Association with Malocclusion in Children: A Transversal Study. Front. Public Health.

[7] Cascone P., Di Paolo C. (2004). Patologia Della Articolazione Temporomandibolare: Dall’eziopatogenesi Alla Terapia.

[8] Rudisch A., Innerhorfer K., Bertram S., Emshoff R. (2001). Magnetic resonance imaging findings of internal derangement and effusion in patients with unilateral temporomandibular joint pain. Oral Surg. Oral Med. Oral Pathol. Oral Radiol. Endod..

[9] Aoyama S., Kino K., Amagasa T., Sakamoto I., Omura K., Honda E., Kobayashi K., Igarashi C., Yoda T. (2002). Clinical and magnetic resonance imaging study of unilateral sideways displacements of temporomandibular joint. J. Med. Dent. Sci..

[10] Taskaya-Yilmaz N., Ogutcen-Toller M. (2001). Magnetic resonance imaging evaluation of temporomandibular joint disc deformities in relation to type of disc displacement. J. Oral Maxillofac. Surg..

[11] Tasaki M.M., Westesson P.L., Kurita K., Mohl N. (1993). Magnetic resonance imaging of the temporomandibular joint. Value of axial images. Oral Surg. Oral Med. Oral Pathol..

[12] Emshoff R., Brandlmaier I., Bertram S., Rudisch A. (2003). Relative odds of temporomandibular joint pain as a function of magnetic resonance imaging findings of internal derangement, osteoarthrosis, effusion, and bone marrow edema. Oral Surg. Oral Med. Oral Pathol. Oral Radiol. Endod..

[13] Emshoff R., Puffer P., Rudisch A., Gassner R. (2000). Temporomandibular joint pain: Relationship to internal derangement type, osteoarthrosis, and synovial fluid mediator level of tumor necrosis factor-alpha. Oral Surg. Oral Med. Oral Pathol. Oral Radiol. Endod..

[14] Sano T. (2000). Recent developments in understanding temporomandibular joint disorders. Part 2: Changes in the retrodiscal tissue. Dentomaxillofac Radiol..

[15] Sano T., Westesson P.L., Larheim T.A., Takagi R. (2000). The association of temporomandibular joint pain with abnormal bone marrow in the mandibular condyle. J. Oral Maxillofac. Surg..

[16] Senga K., Mizutani H., Kobayashi M., Ueda M. (1999). Ultrastructural study on adhesions in internal derangement of the temporomandibular joint. J. Oral Maxillofac. Surg..

[17] Fernandes Pinheiro P., Andrade Da Cunha D., Genuíno Dourado Filho M., Salvetti Cavalcanti Caldas A., Myriam Aragão Melo T., Justino Da Silva H. (2012). The Use of Electrognathography in Jaw Movement Research: A Literature Review. CRANIO®.

[18] Schmitter M., Zahran M., Duc J.-M.P., Henschel V., Rammelsberg P. (2005). Conservative Therapy in Patients With Anterior Disc Displacement Without Reduction Using 2 Common Splints: A Randomized Clinical Trial. J. Oral Maxillofac. Surg..

[19] Ohnuki T., Fukuda M., Nakata A., Nagai H., Takahashi T., Sasano T., Miyamoto Y. (2006). Evaluation of the Position, Mobility, and Morphology of the Disc by MRI before and after Four Different Treatments for Temporomandibular Joint Disorders. Dentomaxillofac. Radiol..

[20] Stiesch-Scholz M., Kempert J., Wolter S., Tschernitschek H., Rossbach A. (2005). Comparative Prospective Study on Splint Therapy of Anterior Disc Displacement without Reduction. J. Oral Rehabil..

[21] Di Paolo C., Falisi G., Panti F., Di Giacomo P., Rampello A. (2020). “RA.DI.CA.” Splint for the Management of the Mandibular Functional Limitation: A Retrospective Study on Patients with Anterior Disc Displacement without Reduction. Int. J. Environ. Res. Public Health.

[22] Di Giacomo P., Di Paolo C., Qorri E., Gatto R., Manes Gravina G., Falisi G. (2022). Conservative Therapies for TMJ Closed Lock: A Randomized Controlled Trial. J. Clin. Med..

[23] Birkett M.A., Day S.J. (1994). Internal Pilot Studies for Estimating Sample Size. Stat. Med..

[24] Drace J.E., Enzmann D.R. (1990). Defining the normal temporomandibular joint: Closed-, partially open-, and open-mouth MR imaging of asymptomatic subjects. Radiology.

[25] Ahmad M., Hollender L., Anderson Q., Kartha K., Ohrbach R.K., Truelove E.L., John M.T., Schiffman E.L. (2009). Research Diagnostic Criteria for Temporomandibular Disorders (RDC/ TMD): Development of image analysis crite- ria and examiner reliability for image analysis. Oral Surg. Oral Med. Oral Pathol. Oral Radiol. Endod..

[26] Sato S., Sakamoto M., Kawamura H., Motegi K. (1999). Long-term changes in clinical signs and symptoms and disc position and morphology in patients with nonreducing disc displacement in the temporomandibular joint. J. Oral Maxillofac. Surg..

[27] Fernandes G., Franco A.L., Goncalves D.A., Speciali J.G., Bigal M.E., Camparis C.M. (2013). Temporomandibular disorders, sleep bruxism, and primary headaches are mutually associated. J. Orofac. Pain.

[28] Goncalves D.A., Camparis C.M., Speciali J.G., Franco A.L., Castanharo S.M., Bigal M.E. (2011). Temporomandibular disorders are differentially associated with headache diagnoses: A controlled study. Clin. J. Pain.

[29] Costa Y.M., Conti P.C., de Faria F.A., Bonjardim L.R. (2017). Temporomandibular disorders and painful comorbidities: Clinical association and underlying mechanisms. Oral Surg. Oral Med. Oral Pathol. Oral Radiol..

[30] van der Meer H.A., Visscher C.M., Engelbert R.H.H., Mulleners W.M., Nijhuis-van der Sanden M.W.G., Speksnijder C.M. (2017). Development and psychometric validation of the headache screening questionnaire—Dutch Version. Musculoskelet. Sci. Pract..

[31] Goncalves D.A.G., Camparis C.M., Speciali J.G., Castanharo S.M., Ujikawa L.T., Lipton R.B., Bigal M.E. (2013). Treatment of comorbid migraine and temporomandibular disorders: A factorial, double-blind, randomized, placebo-controlled study. J. Orofac. Pain.

[32] Maluf S., Moreno B., Crivello O., Cabral C., Bortolotti G., Marques A. (2010). Global postural reeducation and static stretching exercises in the treatment of myogenic temporomandibular disorders: A randomized study. J. Manip. Physiol. Ther..

[33] von Piekartz H., Rösner C., Batz A., Hall T., Ballenberger N. (2020). Bruxism, temporomandibular dysfunction and cervical impairments in females—Results from an observational study. Musculoskelet. Sci. Pract..

